# Supplementary material for: Combined Transcriptome Analysis Reveals the Ovule Abortion Regulatory Mechanisms in the Female Sterile Line of Pinus tabuliformis Carr
Source: Int J Mol Sci. 2021 Mar 19;22(6):3138. doi: 10.3390/ijms22063138 (PMC8003466; doi:10.3390/ijms22063138)
Supplement: Supplementary file 1 [file ijms-22-03138-s001.pdf]

Supplementary Materials

Combined Transcriptome Analysis Reveals the Ovule Abortion Regulatory Mechanisms in the Female Sterile Line of *Pinus tabuliformis* Carr.

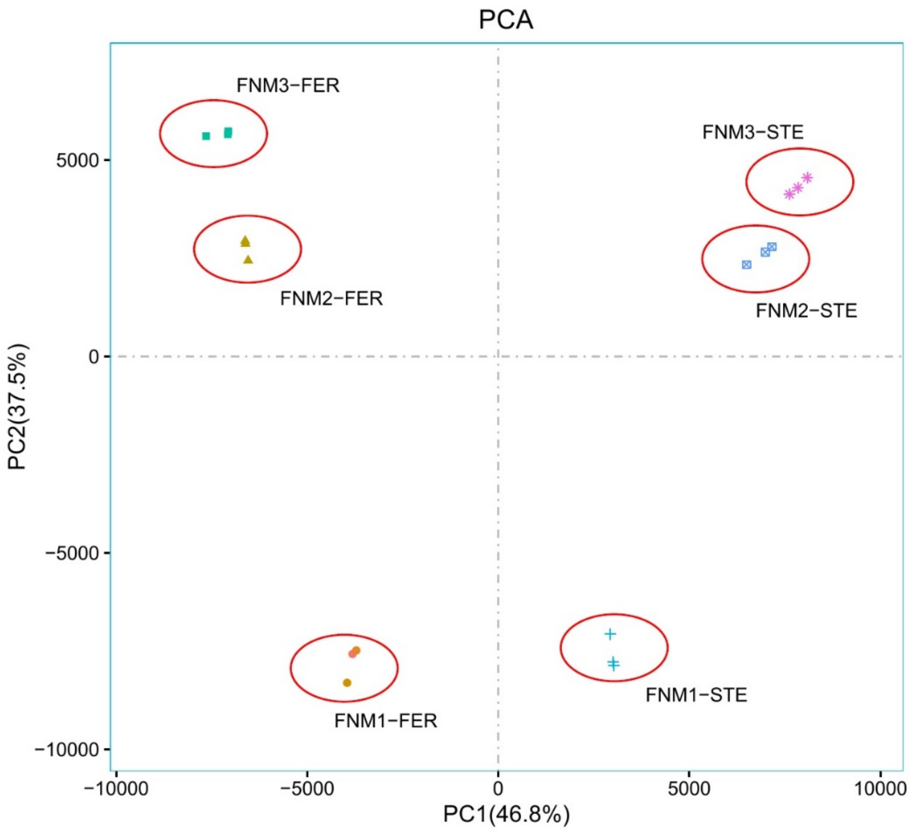

Figure S1. Principal component analysis of the RNA-Seq data.

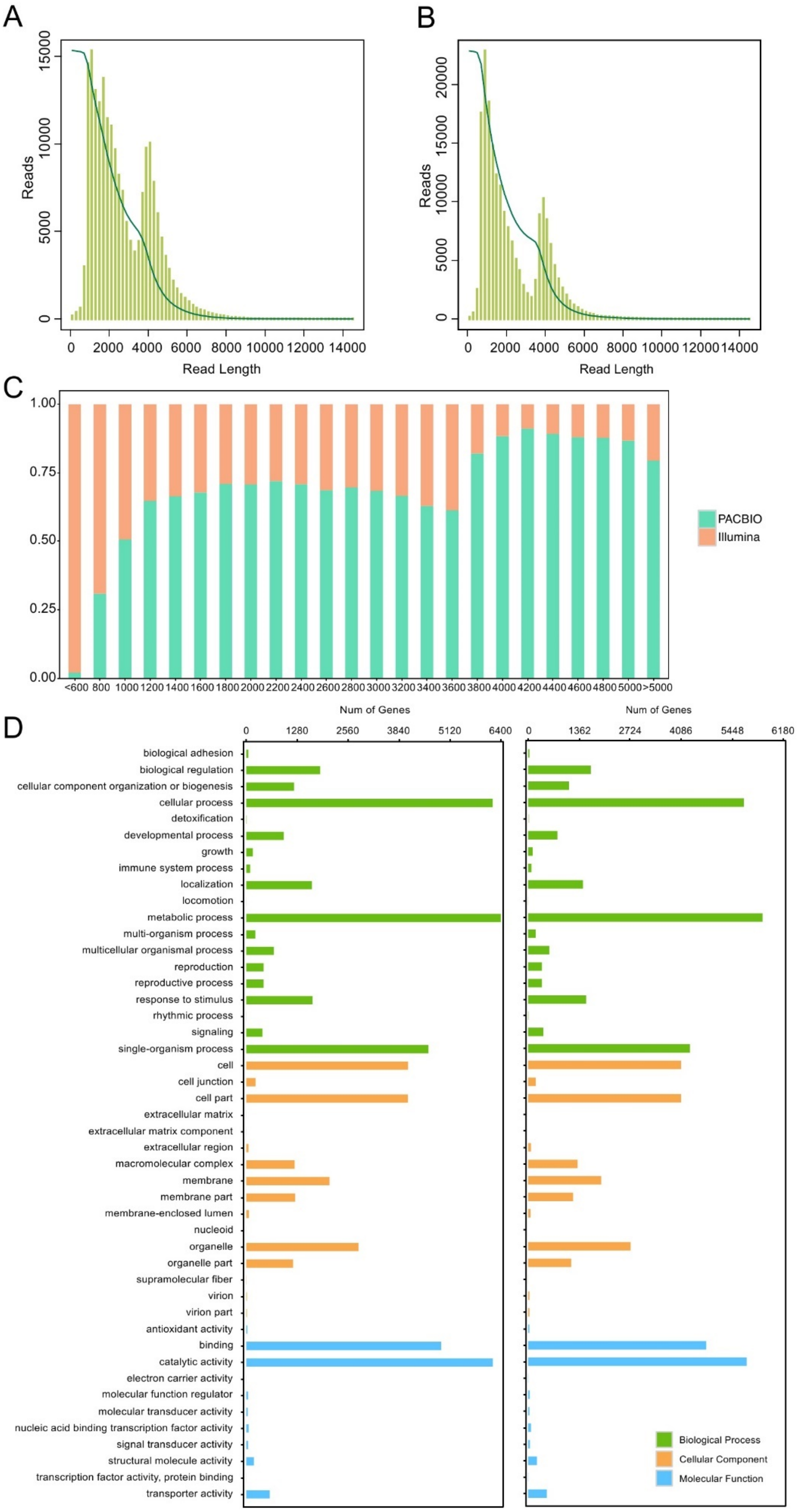

Figure S2. Length distribution of FLNC, FER (**A**), STE (**B**); comparison of transcript length distribution from different sequencing platforms (**C**); GO classification of full-length transcriptomes of FER and STE ovules in *P. tabuliformis* (**D**).

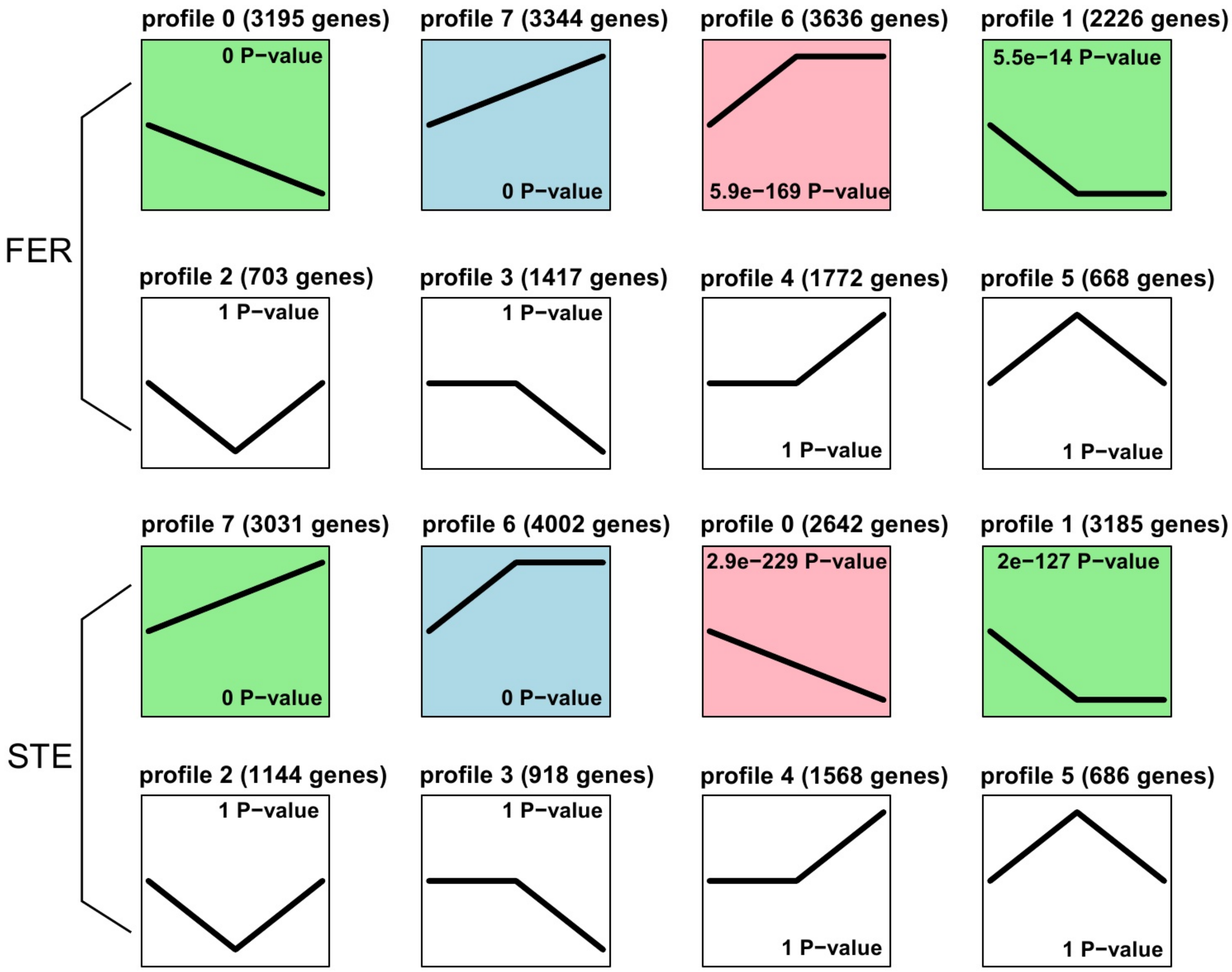

Figure S3. The expression patterns of DEGs analyzed by model profile.

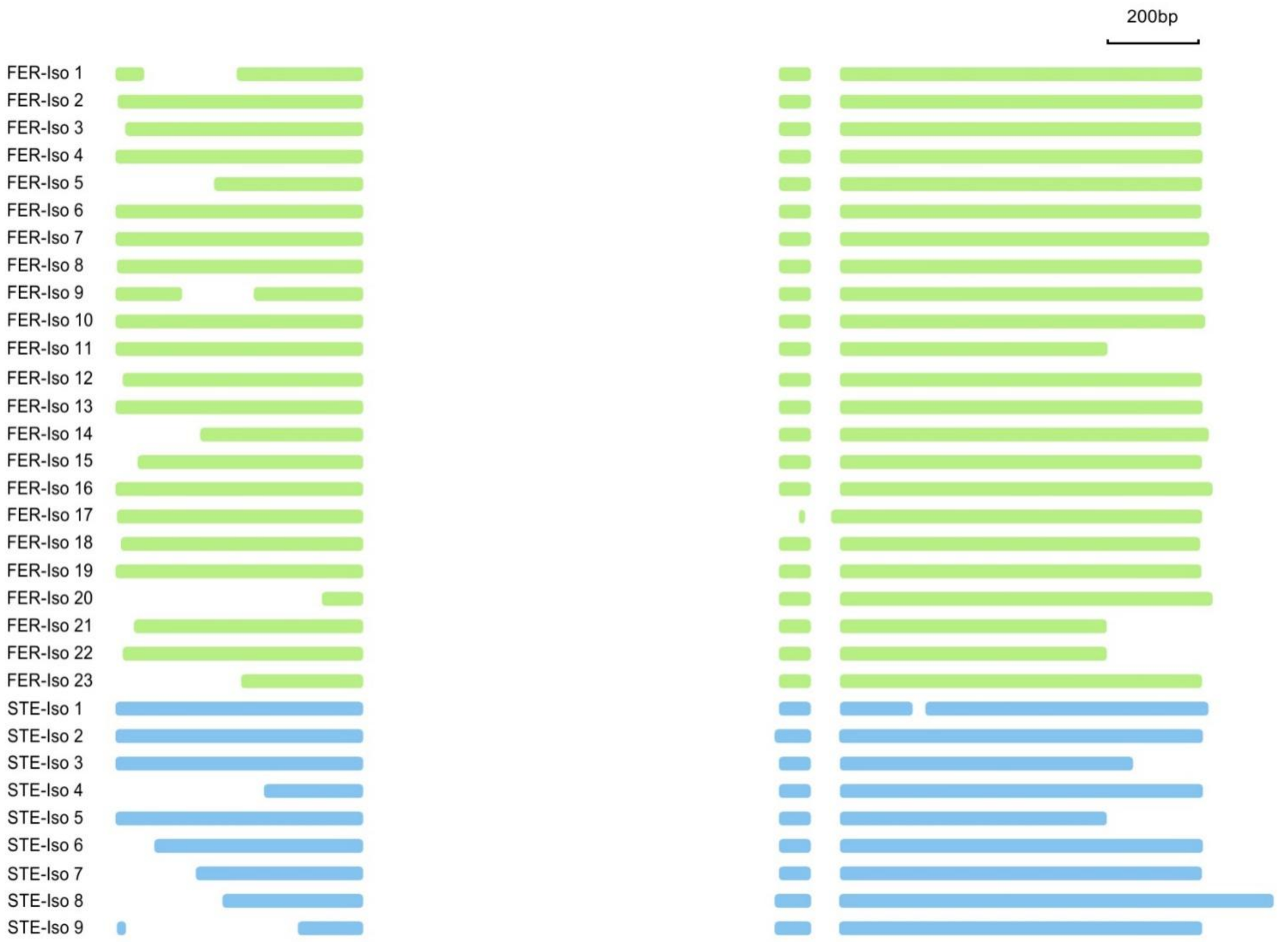

Figure S4. The model graph of *TUBA* generating different transcript isoforms of A3 splicing detected in FER and STE.

Table S1. Summary of SMRT sequencing.

Sample	Total Number	Total Length (bp)	Maximum Length (bp)	Minimum Length (bp)	Average Length (bp)	N50 Length (bp)	GC Content
FER	40828	112127427	9297	131	2746.34	3621	41.59%
STE	44195	98231600	10678	150	2222.69	3322	41.40%

Table S2. KEGG pathway enrichment of DEGs of FER based on SMRT.

	Pathway	All Genes With Pathway Annotation (10062)	Pathway ID
1	Metabolic pathways	3367 (33.46%)	ko01100
2	Biosynthesis of secondary metabolites	1971 (19.59%)	ko01110
3	Biosynthesis of antibiotics	1118 (11.11%)	ko01130
4	Microbial metabolism in diverse environments	956 (9.5%)	ko01120
5	RNA transport	730 (7.26%)	ko03013
6	Protein processing in endoplasmic reticulum	711 (7.07%)	ko04141
7	Carbon metabolism	695 (6.91%)	ko01200
8	Spliceosome	692 (6.88%)	ko03040
9	Biosynthesis of amino acids	607 (6.03%)	ko01230
10	Ribosome	528 (5.25%)	ko03010
11	mRNA surveillance pathway	465 (4.62%)	ko03015
12	Endocytosis	447 (4.44%)	ko04144
13	Plant hormone signal transduction	446 (4.43%)	ko04075

14	Ubiquitin mediated proteolysis	438 (4.35%)	ko04120
15	Glycolysis/Gluconeogenesis	406 (4.03%)	ko00010
16	RNA degradation	368 (3.66%)	ko03018
17	Starch and sucrose metabolism	360 (3.58%)	ko00500
18	Purine metabolism	357 (3.55%)	ko00230
19	Ribosome biogenesis in eukaryotes	321 (3.19%)	ko03008
20	Pyruvate metabolism	292 (2.9%)	ko00620
21	Phagosome	274 (2.72%)	ko04145
22	Plant-pathogen interaction	250 (2.48%)	ko04626
23	Pyrimidine metabolism	249 (2.47%)	ko00240
24	Amino sugar and nucleotide sugar metabolism	248 (2.46%)	ko00520
25	Oxidative phosphorylation	243 (2.42%)	ko00190
26	Cysteine and methionine metabolism	232 (2.31%)	ko00270
27	Fatty acid metabolism	230 (2.29%)	ko01212
28	Proteasome	215 (2.14%)	ko03050
29	Carbon fixation in photosynthetic organisms	200 (1.99%)	ko00710
30	Fatty acid degradation	195 (1.94%)	ko00071
31	Peroxisome	194 (1.93%)	ko04146
32	Citrate cycle (TCA cycle)	187 (1.86%)	ko00020
33	Aminoacyl-tRNA biosynthesis	183 (1.82%)	ko00970
34	Glyoxylate and dicarboxylate metabolism	173 (1.72%)	ko00630
35	Phenylpropanoid biosynthesis	166 (1.65%)	ko00940
36	Galactose metabolism	164 (1.63%)	ko00052
37	Glutathione metabolism	162 (1.61%)	ko00480
38	Glycine, serine and threonine metabolism	161 (1.6%)	ko00260
39	Glycerophospholipid metabolism	160 (1.59%)	ko00564
40	Nucleotide excision repair	157 (1.56%)	ko03420
41	DNA replication	148 (1.47%)	ko03030
42	Fatty acid biosynthesis	139 (1.38%)	ko00061
43	2-Oxocarboxylic acid metabolism	138 (1.37%)	ko01210
44	alpha-Linolenic acid metabolism	135 (1.34%)	ko00592
45	Alanine, aspartate and glutamate metabolism	132 (1.31%)	ko00250
46	Arginine and proline metabolism	132 (1.31%)	ko00330
47	Fructose and mannose metabolism	131 (1.3%)	ko00051
48	Tyrosine metabolism	127 (1.26%)	ko00350
49	Inositol phosphate metabolism	119 (1.18%)	ko00562
50	Circadian rhythm - plant	119 (1.18%)	ko04712
51	beta-Alanine metabolism	116 (1.15%)	ko00410
52	Pentose phosphate pathway	113 (1.12%)	ko00030
53	ABC transporters	112 (1.11%)	ko02010
54	Ascorbate and aldarate metabolism	110 (1.09%)	ko00053
55	RNA polymerase	110 (1.09%)	ko03020
56	Phosphatidylinositol signaling system	110 (1.09%)	ko04070
57	Pentose and glucuronate interconversions	109 (1.08%)	ko00040
58	Lysine degradation	106 (1.05%)	ko00310
59	Mismatch repair	104 (1.03%)	ko03430
60	Valine, leucine and isoleucine degradation	101 (1%)	ko00280
61	Propanoate metabolism	99 (0.98%)	ko00640
62	Phenylalanine metabolism	98 (0.97%)	ko00360
63	Protein export	95 (0.94%)	ko03060
64	Terpenoid backbone biosynthesis	94 (0.93%)	ko00900
65	Flavonoid biosynthesis	93 (0.92%)	ko00941

66	Glycerolipid metabolism	90 (0.89%)	ko00561
67	Basal transcription factors	88 (0.87%)	ko03022
68	Regulation of autophagy	83 (0.82%)	ko04140
69	Phenylalanine, tyrosine and tryptophan biosynthesis	80 (0.8%)	ko00400
70	Tryptophan metabolism	77 (0.77%)	ko00380
71	Homologous recombination	74 (0.74%)	ko03440
72	Biosynthesis of unsaturated fatty acids	73 (0.73%)	ko01040
73	Cyanoamino acid metabolism	71 (0.71%)	ko00460
74	N-Glycan biosynthesis	71 (0.71%)	ko00510
75	Sphingolipid metabolism	69 (0.69%)	ko00600
76	Pantothenate and CoA biosynthesis	68 (0.68%)	ko00770
77	Base excision repair	68 (0.68%)	ko03410
78	Butanoate metabolism	62 (0.62%)	ko00650
79	Porphyrin and chlorophyll metabolism	61 (0.61%)	ko00860
80	Arginine biosynthesis	60 (0.6%)	ko00220
81	Histidine metabolism	60 (0.6%)	ko00340
82	Ether lipid metabolism	60 (0.6%)	ko00565
83	Tropane, piperidine and pyridine alkaloid biosynthesis	57 (0.57%)	ko00960
84	Selenocompound metabolism	54 (0.54%)	ko00450
85	One carbon pool by folate	51 (0.51%)	ko00670
86	Isoquinoline alkaloid biosynthesis	49 (0.49%)	ko00950
87	Steroid biosynthesis	47 (0.47%)	ko00100
88	SNARE interactions in vesicular transport	47 (0.47%)	ko04130
89	Valine, leucine and isoleucine biosynthesis	46 (0.46%)	ko00290
90	Nitrogen metabolism	45 (0.45%)	ko00910
91	Photosynthesis	38 (0.38%)	ko00195
92	Ubiquinone and other terpenoid-quinone biosynthesis	37 (0.37%)	ko00130
93	Stilbenoid, diarylheptanoid and gingerol biosynthesis	37 (0.37%)	ko00945
94	Fatty acid elongation	36 (0.36%)	ko00062
95	Sulfur metabolism	34 (0.34%)	ko00920
96	Taurine and hypotaurine metabolism	33 (0.33%)	ko00430
97	Other glycan degradation	30 (0.3%)	ko00511
98	Lysine biosynthesis	28 (0.28%)	ko00300
99	Nicotinate and nicotinamide metabolism	28 (0.28%)	ko00760
100	Synthesis and degradation of ketone bodies	26 (0.26%)	ko00072
101	Other types of O-glycan biosynthesis	26 (0.26%)	ko00514
102	Cutin, suberine and wax biosynthesis	24 (0.24%)	ko00073
103	Biotin metabolism	24 (0.24%)	ko00780
104	Linoleic acid metabolism	23 (0.23%)	ko00591
105	Vitamin B6 metabolism	23 (0.23%)	ko00750
106	Flavone and flavonol biosynthesis	23 (0.23%)	ko00944
107	Monobactam biosynthesis	22 (0.22%)	ko00261
108	Folate biosynthesis	22 (0.22%)	ko00790
109	Limonene and pinene degradation	22 (0.22%)	ko00903
110	Arachidonic acid metabolism	20 (0.2%)	ko00590
111	Thiamine metabolism	19 (0.19%)	ko00730
112	Non-homologous end-joining	19 (0.19%)	ko03450
113	C5-Branched dibasic acid metabolism	18 (0.18%)	ko00660
114	Glycosylphosphatidylinositol(GPI)-anchor biosynthesis	16 (0.16%)	ko00563
115	Sulfur relay system	15 (0.15%)	ko04122
116	Glycosphingolipid biosynthesis—globo series	14 (0.14%)	ko00603

117	Degradation of aromatic compounds	14 (0.14%)	ko01220
118	Glycosaminoglycan degradation	12 (0.12%)	ko00531
119	Carotenoid biosynthesis	11 (0.11%)	ko00906
120	Photosynthesis - antenna proteins	8 (0.08%)	ko00196
121	Brassinosteroid biosynthesis	7 (0.07%)	ko00905
122	Lipoic acid metabolism	6 (0.06%)	ko00785
123	Zeatin biosynthesis	6 (0.06%)	ko00908
124	Glycosphingolipid biosynthesis—ganglio series	5 (0.05%)	ko00604
125	Sesquiterpenoid and triterpenoid biosynthesis	5 (0.05%)	ko00909
126	Caffeine metabolism	4 (0.04%)	ko00232
127	Diterpenoid biosynthesis	4 (0.04%)	ko00904
128	Riboflavin metabolism	2 (0.02%)	ko00740
129	Anthocyanin biosynthesis	1 (0.01%)	ko00942

Table S3. KEGG pathway enrichment of DEGs of STE based on SMRT.

	Pathway	All Genes With Pathway Annotation (11185)	Pathway ID
1	Metabolic pathways	4116 (36.8%)	ko01100
2	Biosynthesis of secondary metabolites	2499 (22.34%)	ko01110
3	Biosynthesis of antibiotics	1379 (12.33%)	ko01130
4	Microbial metabolism in diverse environments	1173 (10.49%)	ko01120
5	Ribosome	925 (8.27%)	ko03010
6	Carbon metabolism	866 (7.74%)	ko01200
7	Biosynthesis of amino acids	772 (6.9%)	ko01230
8	Spliceosome	736 (6.58%)	ko03040
9	Protein processing in endoplasmic reticulum	725 (6.48%)	ko04141
10	RNA transport	695 (6.21%)	ko03013
11	Glycolysis/Gluconeogenesis	558 (4.99%)	ko00010
12	Endocytosis	472 (4.22%)	ko04144
13	Ubiquitin mediated proteolysis	454 (4.06%)	ko04120
14	Plant hormone signal transduction	424 (3.79%)	ko04075
15	mRNA surveillance pathway	400 (3.58%)	ko03015
16	Starch and sucrose metabolism	384 (3.43%)	ko00500
17	RNA degradation	368 (3.29%)	ko03018
18	Purine metabolism	355 (3.17%)	ko00230
19	Ribosome biogenesis in eukaryotes	347 (3.1%)	ko03008
20	Cysteine and methionine metabolism	323 (2.89%)	ko00270
21	Pyruvate metabolism	310 (2.77%)	ko00620
22	Oxidative phosphorylation	301 (2.69%)	ko00190
23	Proteasome	269 (2.41%)	ko03050
24	Plant-pathogen interaction	264 (2.36%)	ko04626
25	Carbon fixation in photosynthetic organisms	260 (2.32%)	ko00710
26	Pyrimidine metabolism	253 (2.26%)	ko00240
27	Phenylpropanoid biosynthesis	246 (2.2%)	ko00940
28	Phagosome	244 (2.18%)	ko04145
29	Fatty acid metabolism	241 (2.15%)	ko01212
30	Citrate cycle (TCA cycle)	238 (2.13%)	ko00020
31	Amino sugar and nucleotide sugar metabolism	232 (2.07%)	ko00520
32	Fatty acid degradation	222 (1.98%)	ko00071
33	Peroxisome	218 (1.95%)	ko04146

34	Glycerophospholipid metabolism	202 (1.81%)	ko00564
35	Glyoxylate and dicarboxylate metabolism	202 (1.81%)	ko00630
36	Glycine, serine and threonine metabolism	194 (1.73%)	ko00260
37	Tyrosine metabolism	187 (1.67%)	ko00350
38	Glutathione metabolism	182 (1.63%)	ko00480
39	2-Oxocarboxylic acid metabolism	178 (1.59%)	ko01210
40	Galactose metabolism	175 (1.56%)	ko00052
41	alpha-Linolenic acid metabolism	167 (1.49%)	ko00592
42	Alanine, aspartate and glutamate metabolism	166 (1.48%)	ko00250
43	Aminoacyl-tRNA biosynthesis	162 (1.45%)	ko00970
44	Fructose and mannose metabolism	161 (1.44%)	ko00051
45	Flavonoid biosynthesis	147 (1.31%)	ko00941
46	Arginine and proline metabolism	143 (1.28%)	ko00330
47	Fatty acid biosynthesis	141 (1.26%)	ko00061
48	beta-Alanine metabolism	141 (1.26%)	ko00410
49	Nucleotide excision repair	139 (1.24%)	ko03420
50	Circadian rhythm - plant	134 (1.2%)	ko04712
51	Pentose phosphate pathway	132 (1.18%)	ko00030
52	Phenylalanine metabolism	130 (1.16%)	ko00360
53	Ascorbate and aldarate metabolism	129 (1.15%)	ko00053
54	Inositol phosphate metabolism	121 (1.08%)	ko00562
55	Valine, leucine and isoleucine degradation	120 (1.07%)	ko00280
56	Glycerolipid metabolism	119 (1.06%)	ko00561
57	Terpenoid backbone biosynthesis	118 (1.05%)	ko00900
58	DNA replication	118 (1.05%)	ko03030
59	Phosphatidylinositol signaling system	116 (1.04%)	ko04070
60	RNA polymerase	115 (1.03%)	ko03020
61	Phenylalanine, tyrosine and tryptophan biosynthesis	112 (1%)	ko00400
62	Protein export	109 (0.97%)	ko03060
63	Pentose and glucuronate interconversions	107 (0.96%)	ko00040
64	Biosynthesis of unsaturated fatty acids	104 (0.93%)	ko01040
65	Mismatch repair	101 (0.9%)	ko03430
66	Lysine degradation	100 (0.89%)	ko00310
67	N-Glycan biosynthesis	96 (0.86%)	ko00510
68	Propanoate metabolism	95 (0.85%)	ko00640
69	Arginine biosynthesis	85 (0.76%)	ko00220
70	Pantothenate and CoA biosynthesis	82 (0.73%)	ko00770
71	ABC transporters	80 (0.72%)	ko02010
72	Regulation of autophagy	79 (0.71%)	ko04140
73	Butanoate metabolism	78 (0.7%)	ko00650
74	Tryptophan metabolism	76 (0.68%)	ko00380
75	Cyanoamino acid metabolism	76 (0.68%)	ko00460
76	Tropane, piperidine and pyridine alkaloid biosynthesis	75 (0.67%)	ko00960
77	Valine, leucine and isoleucine biosynthesis	71 (0.63%)	ko00290
78	Selenocompound metabolism	70 (0.63%)	ko00450
79	Ether lipid metabolism	70 (0.63%)	ko00565
80	Basal transcription factors	70 (0.63%)	ko03022
81	Porphyrin and chlorophyll metabolism	67 (0.6%)	ko00860
82	Homologous recombination	67 (0.6%)	ko03440
83	Sphingolipid metabolism	66 (0.59%)	ko00600
84	Base excision repair	64 (0.57%)	ko03410
85	Isoquinoline alkaloid biosynthesis	62 (0.55%)	ko00950

86	Photosynthesis	56 (0.5%)	ko00195
87	Histidine metabolism	55 (0.49%)	ko00340
88	Sulfur metabolism	55 (0.49%)	ko00920
89	Nitrogen metabolism	54 (0.48%)	ko00910
90	Biotin metabolism	52 (0.46%)	ko00780
91	SNARE interactions in vesicular transport	51 (0.46%)	ko04130
92	Steroid biosynthesis	49 (0.44%)	ko00100
93	Stilbenoid, diarylheptanoid and gingerol biosynthesis	46 (0.41%)	ko00945
94	One carbon pool by folate	44 (0.39%)	ko00670
95	Ubiquinone and other terpenoid-quinone biosynthesis	43 (0.38%)	ko00130
96	Taurine and hypotaurine metabolism	37 (0.33%)	ko00430
97	Degradation of aromatic compounds	37 (0.33%)	ko01220
98	Arachidonic acid metabolism	35 (0.31%)	ko00590
99	Nicotinate and nicotinamide metabolism	33 (0.3%)	ko00760
100	Linoleic acid metabolism	31 (0.28%)	ko00591
101	Lysine biosynthesis	30 (0.27%)	ko00300
102	Fatty acid elongation	28 (0.25%)	ko00062
103	Monobactam biosynthesis	28 (0.25%)	ko00261
104	Thiamine metabolism	28 (0.25%)	ko00730
105	Other types of O-glycan biosynthesis	27 (0.24%)	ko00514
106	Limonene and pinene degradation	27 (0.24%)	ko00903
107	Other glycan degradation	25 (0.22%)	ko00511
108	Synthesis and degradation of ketone bodies	24 (0.21%)	ko00072
109	C5-Branched dibasic acid metabolism	23 (0.21%)	ko00660
110	Vitamin B6 metabolism	23 (0.21%)	ko00750
111	Cutin, suberine and wax biosynthesis	21 (0.19%)	ko00073
112	Glycosylphosphatidylinositol(GPI)-anchor biosynthesis	20 (0.18%)	ko00563
113	Folate biosynthesis	20 (0.18%)	ko00790
114	Carotenoid biosynthesis	20 (0.18%)	ko00906
115	Flavone and flavonol biosynthesis	17 (0.15%)	ko00944
116	Non-homologous end-joining	16 (0.14%)	ko03450
117	Sulfur relay system	16 (0.14%)	ko04122
118	Glycosaminoglycan degradation	15 (0.13%)	ko00531
119	Brassinosteroid biosynthesis	15 (0.13%)	ko00905
120	Zeatin biosynthesis	15 (0.13%)	ko00908
121	Sesquiterpenoid and triterpenoid biosynthesis	15 (0.13%)	ko00909
122	Glycosphingolipid biosynthesis - globo series	14 (0.13%)	ko00603
123	Photosynthesis - antenna proteins	13 (0.12%)	ko00196
124	Diterpenoid biosynthesis	12 (0.11%)	ko00904
125	Caffeine metabolism	7 (0.06%)	ko00232
126	Riboflavin metabolism	4 (0.04%)	ko00740
127	Lipoic acid metabolism	3 (0.03%)	ko00785
128	Glycosphingolipid biosynthesis - ganglio series	2 (0.02%)	ko00604
129	Monoterpenoid biosynthesis	1 (0.01%)	ko00902

Table S4. KEGG pathway enrichment of DEGs of upregulated in FER also downregulated in STE.

	Pathway	DEGs Genes with Pathway Annotation (4)	All genes With Pathway Annotation (20069)	Pvalue	Qvalue	Pathway ID
1	Pentose phosphate pathway	1 (25%)	226 (1.13%)	0.04429	0.22261	ko00030
2	Fructose and mannose metabolism	1 (25%)	272 (1.36%)	0.05313	0.22261	ko00051
3	Galactose metabolism	1 (25%)	317 (1.58%)	0.06171	0.22261	ko00052
4	Aminoacyl-tRNA biosynthesis	1 (25%)	327 (1.63%)	0.0636	0.22261	ko00970
5	Phagosome	1 (25%)	475 (2.37%)	0.09137	0.25584	ko04145
6	RNA degradation	1 (25%)	699 (3.48%)	0.13222	0.30851	ko03018
7	Glycolysis/Gluconeogenesis	1 (25%)	902 (4.49%)	0.16803	0.33606	ko00010
8	Biosynthesis of amino acids	1 (25%)	1287 (6.41%)	0.23289	0.36196	ko01230
9	Ribosome	1 (25%)	1359 (6.77%)	0.24459	0.36196	ko03010
10	Carbon metabolism	1 (25%)	1446 (7.21%)	0.25854	0.36196	ko01200
11	Microbial metabolism in diverse environments	1 (25%)	1991 (9.92%)	0.34161	0.43477	ko01120
12	Biosynthesis of antibiotics	1 (25%)	2319 (11.56%)	0.38811	0.4528	ko01130
13	Biosynthesis of secondary metabolites	1 (25%)	4201 (20.93%)	0.6092	0.65607	ko01110
14	Metabolic pathways	1 (25%)	7058 (35.17%)	0.82337	0.82337	ko01100

Table S5. KEGG pathway enrichment of DEGs of upregulated in STE also downregulated in FER.

	Pathway	DEGs Genes With Pathway Annotation (25)	All Genes With Pathway Annotation (20069)	Pvalue	Qvalue	Pathway ID
1	Phenylpropanoid biosynthesis	7 (28%)	395 (1.97%)	0	9E-06	ko00940
2	Metabolic pathways	19 (76%)	7058 (35.17%)	3.7E-05	0.00044	ko01100
3	Starch and sucrose metabolism	6 (24%)	703 (3.5%)	0.00018	0.00145	ko00500
4	Biosynthesis of secondary metabolites	12 (48%)	4201 (20.93%)	0.00231	0.01387	ko01110
5	Phenylalanine, tyrosine and tryptophan biosynthesis	2 (8%)	182 (0.91%)	0.0214	0.10272	ko00400
6	Taurine and hypotaurine metabolism	1 (4%)	68 (0.34%)	0.0814	0.32559	ko00430
7	Amino sugar and nucleotide sugar metabolism	2 (8%)	454 (2.26%)	0.10897	0.33576	ko00520
8	Nitrogen metabolism	1 (4%)	95 (0.47%)	0.11192	0.33576	ko00910
9	Butanoate metabolism	1 (4%)	134 (0.67%)	0.1543	0.41145	ko00650
10	Phosphatidylinositol signaling system	1 (4%)	219 (1.09%)	0.24003	0.51106	ko04070
11	Inositol phosphate metabolism	1 (4%)	230 (1.15%)	0.25049	0.51106	ko00562
12	beta-Alanine metabolism	1 (4%)	245 (1.22%)	0.26454	0.51106	ko00410
13	Endocytosis	2 (8%)	855 (4.26%)	0.28864	0.51106	ko04144
14	Alanine, aspartate and glutamate metabolism	1 (4%)	282 (1.41%)	0.29812	0.51106	ko00250
15	Citrate cycle (TCA cycle)	1 (4%)	389 (1.94%)	0.38715	0.61944	ko00020
16	Oxidative phosphorylation	1 (4%)	503 (2.51%)	0.47004	0.68141	ko00190
17	Biosynthesis of amino acids	2 (8%)	1287 (6.41%)	0.48267	0.68141	ko01230
18	Spliceosome	2 (8%)	1367 (6.81%)	0.51547	0.68729	ko03040
19	Biosynthesis of antibiotics	3 (12%)	2319 (11.56%)	0.56427	0.71276	ko01130
20	Plant hormone signal transduction	1 (4%)	800 (3.99%)	0.63854	0.74087	ko04075
21	mRNA surveillance pathway	1 (4%)	821 (4.09%)	0.64826	0.74087	ko03015
22	Microbial metabolism in diverse environments	2 (8%)	1991 (9.92%)	0.72474	0.79063	ko01120
23	Protein processing in endoplasmic reticulum	1 (4%)	1338 (6.67%)	0.822	0.82366	ko04141
24	RNA transport	1 (4%)	1345 (6.7%)	0.82366	0.82366	ko03013

Table S6. Genes list with obvious different expression levels in FER and STE.

Symbol	FER-FNM1-1_rpkm	FER-FNM1-2_rpkm	FER-FNM1-3_rpkm	FER-FNM2-1_rpkm	FER-FNM2-2_rpkm	FER-FNM2-3_rpkm	FER-FNM3-1_rpkm	FER-FNM3-2_rpkm	FER-FNM3-3_rpkm	STE-FNM1-1_rpkm	STE-FNM1-2_rpkm	STE-FNM1-3_rpkm	STE-FNM2-1_rpkm	STE-FNM2-2_rpkm	STE-FNM2-3_rpkm	STE-FNM3-1_rpkm	STE-FNM3-2_rpkm	STE-FNM3-3_rpkm
PFK2	40.45	30.98	35.29	92.41	70.87	110.28	140.7	210.05	115.75	150.57	12.77	14.1	3.14	3.73	5.97	6.16	7.98	7.49
SUS2	17.06	27.19	16.43	16.81	11.24	10.36	9.06	8.51	5.25	2.31	2.35	2.2	8.99	10.94	12.89	5.17	8.86	6.81
SUS	54.85	90.14	51.07	48.92	39.44	34.02	21.84	31.52	19.89	7.01	6.12	9.37	44.54	45.2	48.23	21.57	32.06	21.4
SUS	26.89	37.46	24.23	16.04	16.19	17.11	9.43	14.69	9.15	2.95	3.54	3.57	17.59	22.24	19.69	8.99	9.59	8.52
SUS	48.91	85.19	49.06	37.55	33.83	29.91	18.65	27.4	17.43	8.27	7.22	9.95	42.38	43.09	45.94	13.61	29.06	18.75
AGPL1	71.98	50.77	47	23.96	19.52	18.29	25.55	23.93	22.39	0	0	0	0.9	0	0	1.78	4.16	0.88
CLV1	5.61	6.85	5.25	2.05	1.64	1.79	2.04	1.48	1.02	0.43	0.43	0.28	0.16	0.79	0.65	1.15	1.3	1.06
CLV1	11.18	12.76	5.3	2.28	1.94	2.02	2.32	2.34	0.93	2.77	2.22	3.38	1.3	2.36	1.33	3.78	3.01	2.84
CLV1	10.32	12.01	9.02	3.29	2.49	3.02	2.87	1.78	1.54	0.49	0.78	0.76	1.02	1.1	1.04	1.71	3.78	2.06
TET18	1.76	1	1.61	2.57	3.14	1.82	8.26	6.56	8.48	11.65	9.86	10.05	3.55	2.58	3.19	2.8	3.49	3.73
RTNLB8	47.37	54.96	38.89	25.74	23.68	23.31	21.28	19.13	15.08	7.81	5.99	6.94	15.2	17.25	16.31	17.93	19.94	16.92
BHLH66	6.12	7.21	7.43	2.99	2.5	2.97	3.53	4.93	3.39	3.88	4.94	4.03	5.54	9.52	7.24	9.57	13.55	10.39
UBQ	114.65	136.38	105.29	50.51	39.12	49.71	78.88	57.93	50.19	10.58	9.01	9.98	9.49	17.9	18.4	14.38	28.58	16.9
SOBIR1	9.23	11.13	5.94	5.16	6.53	6.78	6.02	3.6	1.51	3.18	2.81	2.82	5.53	6.5	7.15	11.44	32.14	22.43
CPI	9.04	10.2	9.34	7.53	7.28	7.56	4.18	4.92	4.48	0	0	0	0.17	0	0.1	0	0.06	0
PER	63	66.15	38.84	20.18	20.78	18.04	10.49	9.86	5.08	1.99	0.51	1.74	2.43	3.92	3.27	15.3	12.25	17.26
PER	22.92	38.87	20.62	5.41	1.52	1.44	13.92	8.9	2.37	1.67	1.21	2.15	8.66	15.76	22.94	2.62	13.99	4.86
PER	240.29	256.72	169.11	62.95	40.3	44.03	17.92	26.02	1.31	24.6	20.29	26.02	31.51	41.48	45.32	37.65	75.11	55.77
STR6	68.08	64.04	56.12	29.88	23.41	25.68	6.33	6.39	4.22	15.45	10.95	10.66	14.96	17.41	20.1	25.28	33.64	22.85

TUBA	69.95	71.72	78.06	212.6	243.56	253.99	719.02	987.65	1347.38	0	0.97	0.22	0	0	0	0	0	0
CYCB	5.83	5.47	5.49	8.2	9.71	8.43	13.47	14.71	19.6	11.49	11.39	10.14	3.5	4.7	5.8	5.25	4.95	4.55
CALS1	0.05	0.04	0.25	0.04	0.08	0	0	0.02	0	37.66	37.13	34.64	69.45	72.47	66.18	73.1	84.51	90.81

Table S7. AS events of *AGPL1*, *bHLH66* and *TUBA* detected in FER and STE.

Gene Name	Seqname	Type	Pos	Strand	Total_transcripts
TUBA	COGENT022181	A3	3048-3139:3048-4093	+	STE.Isoform0011529,FER.Isoform0013504,FER.Isoform0000665
TUBA	COGENT022181	RI	3139:3839-3891:4281	+	FER.Isoform0013504,STE.Isoform0025073
AGPL1	COGENT005093	RI	1074:1128-3269:3362	+	FER.Isoform0034717,FER.Isoform0016344, FER.Isoform0023129,STE.Isoform0026880, FER.Isoform0003271
bHLH66	COGENT005831	RI	1:68-122:1502	+	FER.Isoform0016909,STE.Isoform0029880

Table S8. The repeat units of SSRs in FER and STE.

Number of Repeat Unit	Di-		Tri-		Tetra-		Penta-		Hexa	
	FER	STE	FER	STE	FER	STE	FER	STE	FER	STE
4–7	1231	1158	2024	1742	461	405	134	130	284	224
8–11	838	836	103	99	2	2	0	0	1	4
12–15	278	301	5	5	0	0	0	0	0	0
16–19	111	133	0	0	0	0	0	0	0	0
20–99	58	57	0	0	0	0	0	0	0	0

Table S9. Primers for qRT-PCR.

Gene ID	Gene Description	Sequence (5′ -> 3′)
Iso_0002325	SUS	CAACCATTACGAGGAAGC<br>CAACTAACACCAAAGCCAC
Iso_0010189	CLV1	GGGTGAACCTGGAAGACG<br>AAGCGGTGAGACAATGAGC
Iso_0014905	UBQ10	GGCGAAGATTCAGGATAAA<br>GAATGTTGTAGTCGGCTAGAGT
Iso_0015412	AGPL1	GGGAGGACATAGGAACAAT<br>TGAATCCAATACCCGACA
Iso_0053653	PER12	GCAGTTGGAGGACCAGAGT<br>AAGCCTTGATGAGGGTGG
Iso_0011552	PFK2	TGCGGTGGTTCTGGCTAT<br>GTTGATGCCTTGGCTCTTG
Iso_0012780	TUBA	TCCAACTCGACCAGTGTAGC<br>AGCAGCCAAGTCCTCACG
Iso_0053321	TET18	GGCTACCCACCAACCTCA<br>CATCCACTGCCACAACGA
Iso_0024626	SOBIR	TTGTTGGGATGCTGTGGC<br>CTGCGTCGGTCTCGTTTT
Reference	EF1	GCAAGGAACTGGAGAAGGAACCC<br>GACGCATGTCCCTCACAGCAA
